# A multidisciplinary approach for the treatment of GIST liver metastasis

**DOI:** 10.1186/1477-7819-6-46

**Published:** 2008-05-09

**Authors:** Pejman Radkani, Marcelo M Ghersi, Juan C Paramo, Thomas W Mesko

**Affiliations:** 1 Department of Surgery, Section of Surgical Oncology, Mount Sinai Medical Center, Miami Beach, Florida, USA

## Abstract

**Background:**

Advanced gastrointestinal stromal tumors (GISTs) can metastasize and recur after a long remission period, resulting in serious morbidity, mortality, and complex management issues.

**Case presentation:**

A 67-year-old woman presented with epigastric fullness, mild jaundice and weight loss with a history of a bowel resection 7 years prior for a primary GIST of the small bowel. The finding of a heterogeneous mass 15.5 cm in diameter replacing most of the left lobe of the liver by ultrasonography and CT, followed by positive cytological studies revealed a metastatic GIST. Perioperative optimization of the patient's nutritional status along with biliary drainage, and portal vein embolization were performed. Imatinib was successful in reducing the tumor size and facilitating surgical resection.

**Conclusion:**

A well-planned multidisciplinary approach should be part of the standard management of advanced or metastatic GIST.

## Background

Gastrointestinal stromal tumors (GISTs) are neoplasms of the gastrointestinal tract. Despite their less aggressive pathologic nature, GISTs can metastasize and recur after a long remission period. Such cases may produce serious morbidity, mortality, and complex management issues for the treating physician. We hereby report the case of a patient who presented with an isolated metastatic GIST to the liver that was successfully treated with a multidisciplinary approach including imatinib therapy, portal vein embolization, and hepatic lobectomy.

## Case presentation

**Figure 1:**
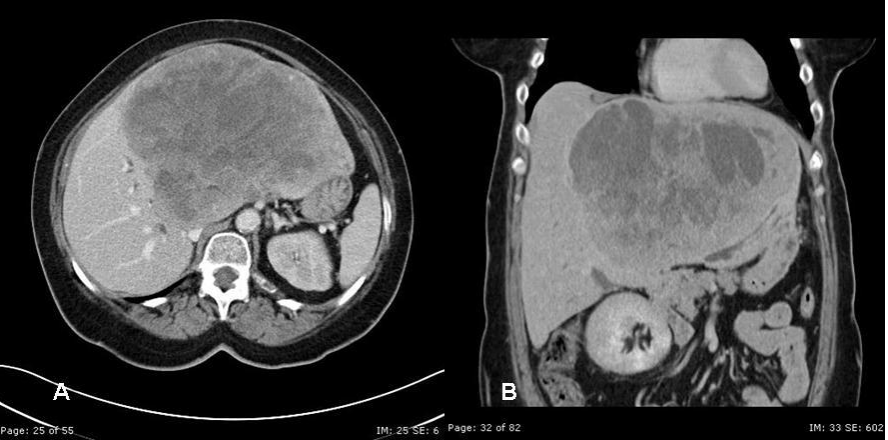
Computerized tomography A) and B); evaluation of the liver demonstrated a large inhomogeneous mass with multiple areas of cystic component within the left lobe of the liver. The mass measured 17.6 × 14 cm. Mild dilatation of the intrahepatic biliary radicals in the right lobe liver.

A 67-year-old woman presented with epigastric fullness, mild jaundice and a 12-pound weight loss over a period of 3 months. The patient had a history of a bowel resection 7 years prior to presentation for an unknown malignancy. On physical examination, her abdomen was soft with a palpable and non-tender mass in the mid-epigastrium. Initial work-up including ultrasonography revealed a large liver lesion, follow-up CT confirmed the presence of a heterogeneous mass 17.5 cm in diameter replacing most of the left lobe of the liver (Figure 1a, 1b) with marked compression of the right biliary tree. Initial Liver function testes showed:

total billirubin: 4, direct billirubin: 3.93, alkaline phosphatase: 942, AST: 124, ALT: 156. The addition laboratory values were within normal limit.

The patient was admitted to the hospital for additional work-up. A percutaneous transhepatic cholangiogram was performed, with placement of a right biliary drainage catheter for decompression. The bilirubin and liver function tests at the day before drainage placement were as follow: total billirubin: 4 direct billirubin: 3.93 ALK: 942 AST: 124 ALT: 156. Two days later the labs were as follow: total billirubin: 3.45 direct billirubin: 3.28, alkaline phosphatase: 788 AST: 117 ALT: 123, and 30 days later was: total billirubin: 0.7 direct billirubin: 0.30 alkaline phosphatase: 130 AST: 28 ALT: 25. A core liver biopsy was also done at the time, which demonstrated atypical spindle cells. Immuno-histochemical studies yielded positive CD117, vimentin and actin stains, all consistent with GIST. It was later established that the patient had previously undergone a small bowel resection for a primary GIST. Upper and lower endoscopy as well as small bowel series were subsequently performed. These revealed no tumors of the GI tract, suggesting the liver mass was a late and isolated metastatic manifestation of the prior GIST tumor.

**Figure 2:**
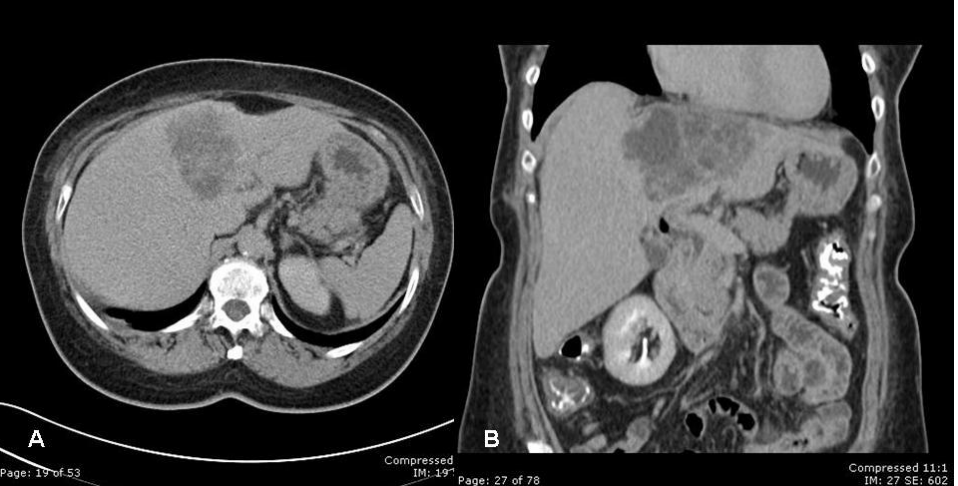
computerized tomography A) and B); mass in the left lobe of the liver has decreased in size with respect to the prior study.

A multidisciplinary and staged treatment course was recommended. Side effects and benefits of using Imatinib drug were considered by our tumor board, and the patient was started at a dose of 600 mg per day to reduce tumor size. The patient was followed regularly for the next few months as an outpatient. Her jaundice resolved and the biliary catheter was successfully removed four months after placement. A significant clinical improvement was noted, with resolution of the patient's initial symptoms and a 7-pound increase in body weight. Frequent abdominal CT scans showed a hepatic mass that diminished in size, but stabilized after 6 months of imatinib therapy at a diameter of 11 cm (Figure 2a, 2b).

The patient then underwent portal vein embolization (PVE) in hopes of promoting hypertrophy of the right lobe and further atrophy of the tumor-laden left hepatic lobe, in preparation for surgical resection.

**Figure 3:**
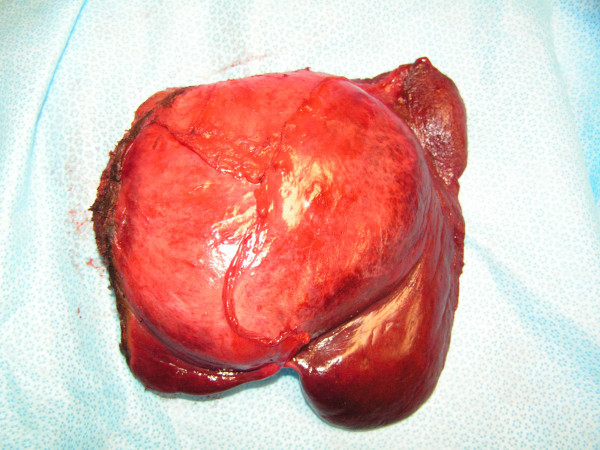
left lobe of the liver, with falciform ligament gallbladder and xiphoid process.

Two months following PVE, while still on imatinib, the patient underwent an uncomplicated left hepatic lobectomy with cholecystectomy (Figure 3). Intraoperative ultrasonography showed a hypertrophied right liver lobe, and a 11 cm tumor involving liver segments 2, 3, 4A an 4B. Pathologic examination corroborated the diagnosis of metastatic GIST with margins of resection free of tumor.

**Figure 4:**
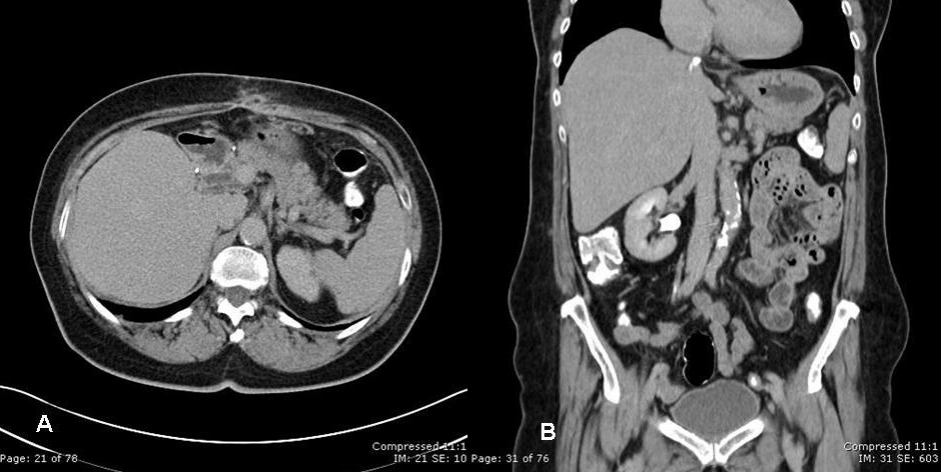
Computerized tomography A) and B); no evidence of metastatic disease.

The patient tolerated the procedure well and was sent home after a 14-day hospitalization. The postoperative course was complicated by the formation of a subhepatic abscess that was successfully treated with drainage catheters and systemic antibiotics. Imatinib was discontinued approximately one month after surgery for a total of one year of therapy. Follow-up CT 6 months after surgery demonstrated no residual neoplastic disease (Figure 4a, 4b). At fourteen-months follow up, the patient was found to be doing very well with no evidence of recurrent disease.

## Discussion

Gastrointestinal stromal tumors are the most common mesenchymal neoplasms of the GI tract. They have an overall incidence of 3000–5000 cases per year in the United States [1-3]. It is thought that these tumors differentiate from intestinal pacemaker cells, also known as interstitial cells of Cajal [1]. They affect mostly males between the ages of 50 and 70, and are usually found incidentally at early stages [1-4]. Large or advanced lesions may present with a variety of clinical findings, including bleeding, abdominal pain, early satiety, bowel obstruction, or perforation.

GISTs are usually detected by endoscopy, CT or MRI performed for abdominal symptoms. The gold standard for diagnosing GISTs is pathological tissue examination, which normally demonstrates atypical splindle cells. A positive stain for CD117 carries a specificity of 95% for these tumors, and will unequivocally establish a diagnosis [1-7].

When GISTs originate in the small bowel, they behave in a more aggressive manner [8]. The most common site for metastases is the liver and the peritoneal cavity, but can also occur in bone, skin, soft tissues, and lymph nodes [5]. Negative prognostic factors for aggressiveness and recurrence include tumor size, a high mitotic index, or an unknown site of origin [9,10]. Recurrence usually occurs 19–26 months after surgery [3,11,12]. For this reason, the National Comprehensive Cancer Network suggests routine follow-up CT scans of the abdomen and pelvis every 3–6 month for the first 3–5 years after resection [7].

The only definitive treatment for GISTs is surgical resection. This can be done laparoscopically in some cases, or with the traditional open approach. The mainstay of surgical therapy in primary or metastatic disease is to achieve a complete resection with negative margins [7]. Conventional chemotherapy and radiation therapy may have minor adjunctive benefits in unresectable or metastatic GISTs [2]. Imatinib mesylate (Gleevec^®^), a selective inhibitor of tyrosine kinase, has revolutionized the management of this disease in recent years. Imatinib has a significant shrinking effect on GISTs, and can be used when primary GISTs have attained a very large size or are in unfavorable locations, increasing the risk of positive resection margins [1]. Imatinib has also become the first line of treatment for recurrent and/or metastatic GISTs, as described for the patient in this case report [13]. Imatinib is generally very well tolerated; and most patients can tolerate treatment without interruption. The more common side effects of Imatinib mesylate include [14,15]: nausea, vomiting, diarrhea, and muscle cramps. It is common to see a decrease in the neutrophil and platelet counts especially during the first month of therapy [16]. The drug should be stopped to allow recovery if the absolute neutrophil count (ANC) falls to <1,000/microL and/or the platelet count to <50,000/microL during the first months of therapy [17]. Our patient had no evidence of these side effects during therapy. More recently, sunitinib malate (Sutent^®^), a multikinase inhibitor has been approved by the Food and Drug Administration for treatment of GISTs that are refractory to imatinib [18].

Resection of GIST liver metastases may be curative when the primary disease has been eradicated and negative surgical resection margins are attained. However, a large tumor burden in the hepatic parenchyma may prohibit resection given the risk of insufficient remaining liver tissue and subsequent postoperative liver failure [19]. An option to counteract this phenomenon is the use of portal vein embolization (PVE) in cases of unilobular involvement of the liver. First used in the 1980s, selective PVE induces atrophy of a selected liver region as well as a compensatory hypertrophy of the remaining liver parenchyma [20-23]. Preoperative PVE is recommended if less than 30% to 40% of normal the liver is expected to remain and be functional after resection [24,25].

## Conclusion

This case, to our best knowledge, represents the first in the literature describing a multidisciplinary approach for the successful management of a large metastasic GIST to the liver. We attribute the success of this case to a well thought out management plan set forth by a dedicated tumor board utilizing advanced and evidence-based therapeutic modalities. Timing and resource utilization were key factors in the management of this patient. Perioperative optimization of the patient's nutritional status and state of health with biliary drainage was helpful. The pharmacologic effect of imatinib reduced the tumor size and improved the surgical resectability. Additionally, PVE facilitated the operation and promoted healthy liver tissue hypertrophy. Lastly, careful operative technique and dedicated follow up allowed for a good surgical outcome in this patient. A well-planned multidisciplinary approach should be part of the standard management of advanced or metastatic GISTs.

## Competing interests

The authors declare that they have no competing interests.

## Authors' contributions

**TM**, **JP **and **MG **designed the study. **PR **carried out the data and bibliographic research and drafted the manuscript. **MG **carried out the picture acquisition, manuscript revision and editing process. **TM **and **JP **did the last manuscript revision and the editing process.
